# Risk of poultry compartments for transmission of Highly Pathogenic Avian Influenza

**DOI:** 10.1371/journal.pone.0207076

**Published:** 2018-11-28

**Authors:** T. J. Hagenaars, G. J. Boender, R. H. M. Bergevoet, H. J. W. van Roermund

**Affiliations:** 1 Wageningen Bioveterinary Research (WBVR), Lelystad, the Netherlands; 2 Wageningen Economic Research, Wageningen, the Netherlands; Tokat Gaziosmanpasa University, TURKEY

## Abstract

When outbreaks of Highly Pathogenic Avian Influenza (HPAI) occur in OIE member countries with until then disease-free status, member countries can use ‘compartmentalisation’. A compartment may be defined as a subset of farms under a common management system, complying with certain stringent surveillance, control and biosecurity measures, and based on that may receive a disease-free status. Based on this disease-free status the compartment is exempted from certain transport restrictions coming into force in case of outbreaks occurring in the country. For deciding whether a candidate compartment is granted official compartment status, it is relevant to assess the additional HPAI transmission risks that would arise due to the exemptions granted. These risks consist of both additional local transmission risks as well as the additional risk of a ‘jump’ of HPAI infection from one poultry area, via the compartment, to another area. Here such risk assessment is carried out using a spatial mathematical model for between-farm transmission in the Netherlands, yielding insight in the roles of compartment composition and contact structure and identify relevant evaluation criteria for granting HPAI compartment status. At the core of this model are transmission probabilities associated with indirect between-farm contacts, e.g. through feed delivery, egg collection and professional visitors. These probabilities were estimated from Dutch epidemic outbreak data in earlier work. The additional risk of a jump of HPAI from one area, via the compartment, to another area is calculated relative to the direct jump risk. The results show that additional transmission risks caused by a compartment to other farms are mainly dependent on the distance of densely populated poultry areas (DPPAs) to the nearest compartment farm. Apart from conditions on these distances, we also recommend specific routing requirements for transport and other movements within the compartment.

## Introduction

Epidemics of highly pathogenic avian influenza (HPAI) can have a large impact on animal welfare, a large economic impact on the poultry industry, and may–due to their zoonotic potential–also have a large impact on public health. In 2003 the Dutch poultry sector was confronted with an HPAI epidemic which started in a densely populated poultry livestock area (DPPA). At the end of the epidemic, 255 farms were infected, 1349 commercial- and 16490 hobby farms were preventively culled to contain the epidemic, and one person died due to an HPAI infection. The total direct costs of the epidemic amounted to € 270 million [1].

Poultry flocks are at a continuous risk of becoming infected by HPAI, mostly through introduction from infection reservoirs in wild birds. In case of an outbreak in an EU member state the infected farms need to be depopulated, transport regulated, additional control measures in a 3 km protection and 10 km surveillance zone (PS zones) set up, dangerous contacts traced, a movement ban and an export ban organised, all according to the requirements of the EU [2]. Additionally, control measures can be taken to further reduce the spread of the infection by pre-emptive culling or emergency vaccination of farms in the vicinity of detected farms [3].

In 2005 the World Organisation for Animal Health (OIE) introduced the concept of compartmentalisation, in the chapter on zoning and regionalisation of its Terrestrial Animal Health Code. As a result, in order to facilitate the continuation of trade in and from unaffected parts of the poultry industry in case of an HPAI epidemic, EU member states can not only use ‘zoning’ but also ‘compartmentalisation’ (Commission Regulation EC No 616/2009, implementing Council Directive 2005/94/EC) [4], [5], [6]. Zoning consists of dividing the country in geographical regions enabling the establishment of infected and infection-free zones during crises. Compartmentalisation means defining certain subsets of farms that reside under a common management system complying with certain stringent surveillance, control and biosecurity measures, such that these can be granted a disease-free status [7], [8], [9], [10]. One of the privileges that may be granted to a compartment is the permission to carry out certain transports within the compartment even from and to compartment farms that are located in an infected area, as long as they are outside protection zones (3 km).

Chapter 4.4 of the OIE Terrestrial Code provides a structured framework for the application and recognition of compartments within countries [6]. Approval requires a detailed and documented biosecurity plan drawn up and implemented for the disease(s) concerned. Scott et al. [8] describes seven factors to guide the identification and documentation process of a compartment which can be used by a country submitting a candidate compartment for EC approval (Commission Regulation EC No 616/2009). Since 2005, concrete initiatives for compartmentalisation have been taken in the EU, Australia, Canada, New Zealand, Thailand and the USA [10], [11]. In the Netherlands, the first application and subsequent approval for a poultry compartment, the ‘VPI’ compartment producing eggs for pharmaceutical purposes, occurred in 2011(application) and 2012 (approval [12]) ‘VPI’ is the abbreviation of the company name to which the compartment farms belong.

In this paper we address the following questions, with specific reference to the example of VPI in the Netherlands:

1. What are the additional transmission risks that a compartment poses during an HPAI epidemic, compared to a situation without compartment, depending on its characteristics? In detail: (a) what is the local HPAI transmission risk from farms within the compartment?, and (b) what is the risk of a ‘jump’ of HPAI via the compartment to other (free) areas in the country? Characteristics considered are: the number of farms within the compartment; whether or not transport of eggs and/or live chicken takes place within the compartment; and the location of the compartment farms in the country (distance to the nearest DPPA). In the Netherlands two DPPAs can be distinguished, both of which are high-risk areas for between-farm transmission [13].
2. Based on these additional risks, what are relevant evaluation criteria for granting the compartment status?

## Materials and methods

### Model structure and approach

A generic model was developed describing HPAI transmission risks between poultry farms in the Netherlands, of which certain specific farms belong to a poultry compartment. As the risk of introduction of HPAI into the country is not part of the research problem, the model focusses on the epidemic period after the first farm in the country was detected as HPAI infected, i.e. after the end of the High-Risk Period (HRP). The model consists of compartment farms and all other non-compartment farms in the country, and is spatial, explicitly including all location coordinates of poultry farms in the Netherlands. The model calculates the HPAI transmission risk between non-compartment farms, from non-compartment farms to compartment farms and vice versa, and between compartment farms.

Even during a standstill situation, when no animal transport is allowed, indirect or ‘neighbourhood’ transmission between farms may still take place, as observed during the 2003 HPAI epidemic in the Netherlands. Non-compartment farms under standstill regulations after the HRP are therefore still ‘connected’ with each other by neighbourhood transmission of HPAI (indirect contact transmission, mostly untraced), although not by transport of live animals (direct contact). Compartment farms are ‘connected’ with each other by direct contacts such as transport of live animals or eggs and also by indirect contacts via e.g. feed deliveries, technical supervisor visits and rendering material collection visits. The probabilities of HPAI transmission by each of these pathways were estimated from the H7N7 HPAI epidemic in the Netherlands in 2003. These probabilities reflect the average biosecurity ‘level’ realized at that time for the processes involved in these pathways. Due to the strict biosecurity measures for the relevant processes taking place within compartments (especially when HPAI is present in the country), the use of these probability estimates from 2003 for processes within the compartment can be viewed as a worst-case assumption. As a model for the indirect between-farm transmission risks we use the so-called spatial transmission kernel based on observed between-herd transmission of HPAI during the 2003 epidemic in the Netherlands [13]. This kernel combines all possible (unknown) transmission routes between poultry farms after the HRP. In our model, this kernel is used to describe neighbourhood transmission among compartment farms, among non-compartment farms and between these. In addition, we explicitly model the risks of direct and known indirect contact events between the compartment farms.

In order to assess the additional transmission risks that a compartment poses, the following two measures of transmission risk were used: (1) the ‘local’ between-farm reproduction number, and (2) the risk of a HPAI transmission ‘jump’ event occurring from one area to another.

### Local between-farm reproduction number *R*_0,*i*_

The local reproduction number *R*_0,*i*_ is associated with farm *i* and defined as the expected number of newly infected farms caused by farm *i* if it became infected and all other (poultry) farms in the country were still susceptible [13]. *R*_*0*,*i*_ is calculated by summing all transmission probabilities *p*_0_ (through neighbourhood transmission during the period after the HRP) from source farm *i* to all possible receiver farms *j*. For source farms outside the compartment(s) this sum is written as follows:

$$R_{0,i}=\sum_{j\neq i}p_{0}\left(r_{ij}\right)$$ (1)

Here *r*_*ij*_ is the straight-line distance from farm *i* to farm *j*. The probability of the between-herd transmission *p*_0_(*r*_*ij*_) during standstill is distance dependent and was described by Boender et al. [13] by

$$p_{0}\left(r_{ij}\right)=1-\exp\left(-\frac{h_{0}T}{1+{\left(\frac{r_{ij}}{r_{0}}\right)}^{\alpha }}\right)$$ (2)

with parameters h_0_ = 0.002 day^-1^, α = 2.1, r_0_ = 1.9 km, and the infectious period of a farm T = 7.5 day, as estimated from the Dutch 2003 HPAI epidemic [13]. Eq 2 describes the so called between-herd transmission kernel for HPAI. This kernel quantifies transmission as the combined result of all (unknown) transmission routes still occurring during standstill (in 2003).

For source farms *m* within the compartment *C* we write:

$$R_{0,m}=\sum_{j\notin C}p_{0}\left(r_{mj}\right)+\sum_{n\in C}p_{c}\left(m\to n\right)$$ (3)

This is the sum of all transmission probabilities *p*_0_ (so caused by neighbourhood transmission only) from source farm *m* to all receiving farms *j* outside the compartment, plus the sum of all transmission probabilities *p*_c_ to all receiving farms *n* within the compartment. These latter probabilities *p*_c_ are caused by both neighbourhood transmission as well as by the between-farm contact events allowed within the compartment. It is assumed that at the spatial scale of the Netherlands, the transmission risks of the latter contact events are independent from the distance between the two compartment farms involved. Mathematically this implies that the probability of transmission *p*_c_ within the compartment is an extension of the indirect transmission probability as follows:

$$p_{c}\left(r_{mn}\right)=1-\exp\left(-\left(\frac{h_{0}}{1+{\left(\frac{r_{mn}}{r_{0}}\right)}^{\alpha }}+\sum_{l}h_{l}\right)T\right)$$ (4)

where *m* is the source farm and *n* is the receiving farm both within the compartment, *r*_*mn*_ is the distance between these farms, and *h*_*l*_ is the transmission rate (per day) for a given between-farm contact event of type *l* within the compartment.

For areas in which farms with *R*_0,*i*_>1 are clustered, the model predicts that there is a risk of a propagating epidemic to occur in that area; for areas where all farms have *R*_0,*i*_<1 only small series of between-farm transmission events are expected. When considering farms within a compartment, note that due to the relatively small number of farms forming the compartment, there is no sharp threshold at *R*_0,*i*_ = 1. This means that then each reduction of *R*_0,*i*_, no matter if it brings *R*_0,*i*_ below one, is important to limit the total number of infected farms in the compartment.

Of particular interest is the difference Δ*R*_0,*i*_ in the local reproduction number between the situation with and without compartment. For source farms *i* outside the compartment(s), the probabilities *p*_0_(*r*_*ij*_) are the same in both situations, and thus the difference is zero in that case:

$$\Delta R_{0,i}=0.$$ (5)

However, for source farms *m* within the compartment *C* the difference is non-zero:

$$\Delta R_{0,m}=\sum_{n\in C}p_{c}\left(m\to n\right).$$ (6)

We note that when in Eq 4 the ‘kernel’ part is much smaller than the contact event transmission rate, i.e. $\frac{h_{0}}{1+{\left(\frac{r_{mn}}{r_{0}}\right)}^{\alpha }}<<\sum_{l}h_{l}$, the distance-independent contact event transmission rate dominates and thus *p*_c_ is in good approximation independent of the distance between the farms (in the compartment). Therefore Eq 6 can be rewritten as:

$$\Delta R_{0,m}=\left(N_{c}-1\right)p_{c}$$ (7)

in which *N*_c_ is the number of farms within the compartment. Only farms with live chicken are considered in *N*_c_, because only these farms can suffer from an HPAI outbreak.

### The additional risk of a jump of HPAI from one area, via the compartment, to another

**Fig 1:**
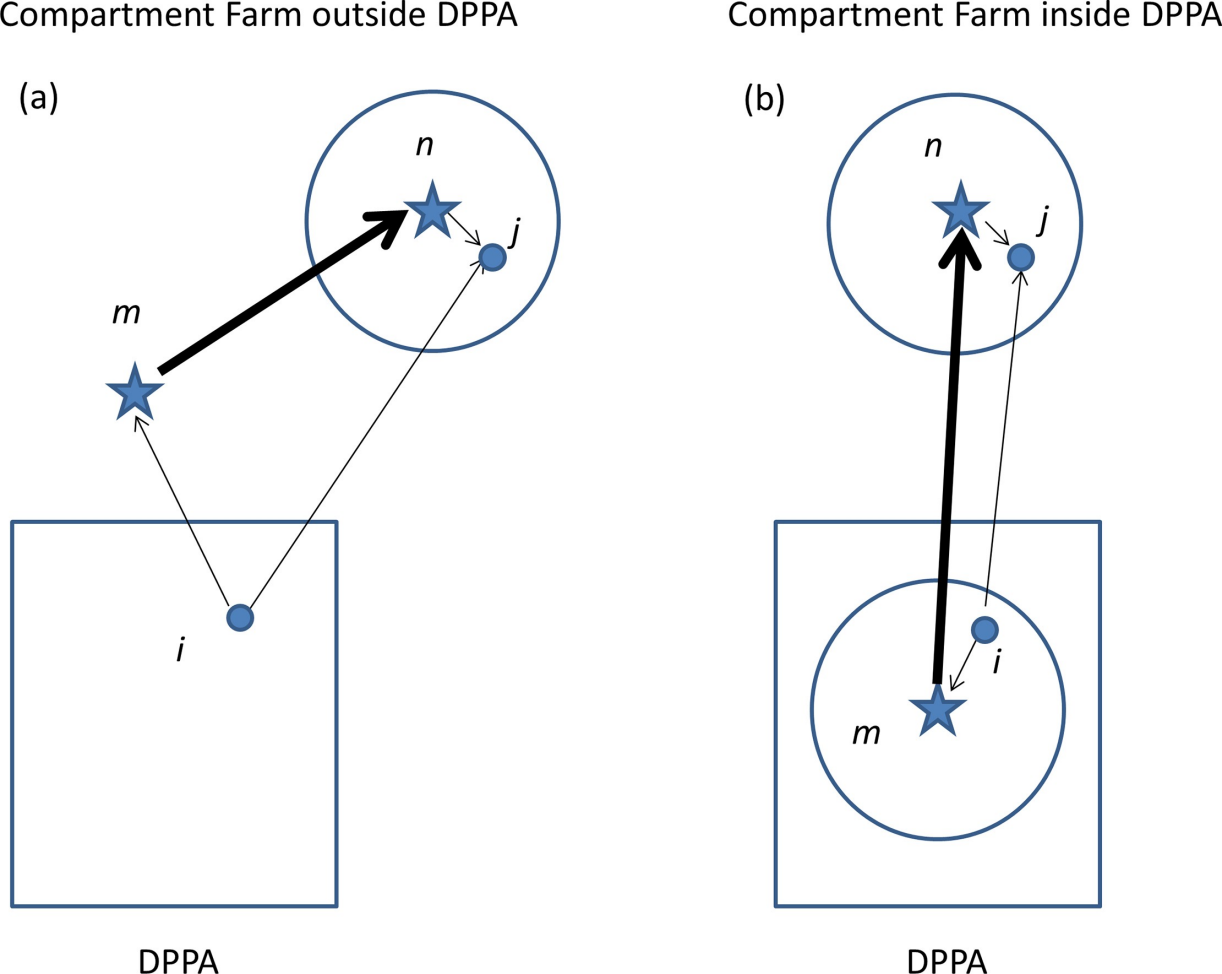
‘Single-event’ transmission from source farm i to receiver farm j, both located outside the compartment (i.e. represented by a dot), compared to ‘triple-event ‘ transmission from source farm i to receiver farm j via farms m and n, both located inside the compartment (i.e. represented by a star). Thick lines represent HPAI transmission by the combination of neighbourhood transmission and by between-herd contact events. Thin lines represent HPAI transmission by neighbourhood transmission only. Circles represent the 10 km surveillance zone around the compartment farms m and n. DPPA means Densely Populated Poultry livestock Area. Shown are the possibilities that the compartment farms (m and n) are situated outside the DPPA (Fig 1A) and that one compartment farm (farm m) is located within the DPPA (Fig 1B).

The allowed transports between compartment farms may produce a link between different areas in the country. This implies that such a compartment constitutes an additional risk of a ‘jump’ of HPAI to another area. To quantify this additional risk, the probability of neighbourhood (‘single-event’) transmission from source farm *i* to receiving farm *j*, both located outside the compartment, is compared to the probability of ‘triple-event’ transmission from source farm *i* to receiving farm *j* via farms *m* and *n* located inside the compartment. This sequence of three transmission events leading to a ‘jump’ is visualized in Fig 1.

The ‘single-event’ transmission is described by the probability *p*_0_ and can be considered as the ‘background’ transmission which is always there at standstill, whereas the ‘triple-event’ transmission via farms in the compartment is the extra risk caused by the presence of the compartment itself. The ‘triple-event’ transmission *p*_t_ is written as the product of three probabilities: transmission from farm *i* outside the compartment to farm *m* inside the compartment, subsequent transmission of farm *m* to farm *n* both inside the compartment, and subsequent transmission of farm *n* to farm *j* outside the compartment:

$$p_{t}\left(i\to m\to n\to j\right)=p_{0}\left(r_{im}\right)p_{c}\left(m\to n\right)p_{0}\left(r_{nj}\right).$$ (8)

The additional transmission jump risk from area A (DPPA in Fig 1) to area B caused by the compartment is evaluated based on *p*_0_ and *p*_t_ as follows. We used 1000 randomly selected sets of farms *i*, *j*, *m*, *n*. Individually these sets are constructed as follows: a random farm *i* in area A was selected. Then, two random compartment farms were selected (*m* and *n*), with farm *n* being located in area B. Subsequently, a random farm (*j*) in area B within a radius of 10 km (the surveillance zone) around farm *n* was selected. The latter 10 km area was chosen because farms in the neighbourhood of a compartment farm are at the highest risk. For each random farm-set the ‘single-event’ transmission probability *p*_0_ from farm *i* to farm *j* was compared with the ‘triple-event’ transmission probability *p*_t_ via compartment farms *m* and *n* by calculating the ratio of the two.

The ratio quantifies how much the ‘triple-event’ transmission contributes relatively to the ‘single-event’ transmission. If this ratio is small, then the contribution of ‘triple-event’ transmission can be neglected. If this ratio is of the order of 1 or higher, the contribution of ‘triple-event’ transmission is about the same as that of the ‘single-event’ transmission.

### Evaluating the effect of distance of the compartment to a DPPA

To evaluate the effect of an increasing distance of a poultry compartment to a densely populated poultry area (DPPA), a similar approach to the one above was used (see Fig 1A). For this calculation a random non-compartment farm *i* was selected in the DPPA (here Gelderse Vallei, an area located in the central part of the Netherlands), a random compartment farm *m* outside the DPPA, a second random compartment farm *n* outside the DPPA and a random non-compartment farm *j* in the 10 km surveillance zone of farm *n*. The ‘single-event’ neighbourhood transmission probability from farm *i* to farm *j* was compared with the ‘triple-event’ transmission probability via compartment farms *m* and *n*. The ratio between the ‘triple-event’ transmission risk to the ‘single-event’ transmission risk was calculated for each selection of farms. Furthermore, the nearest distance of the compartment farm *m* to a farm in the DPPA was determined. This selection process was repeated 10,000 times, leading to a set of pairs {distance, ratio}. Based on this set a rolling average plot was calculated with a 5-km wide averaging window. The rolling average of the mean, the 5% percentile and 95% percentile is presented.

### Poultry farm location data

Poultry farm location data from 2008 were used, representative of the situation when the example compartment of VPI (compartment status granted in 2011) was discussed in the Netherlands. The Dutch poultry situation in that year involved 2771 commercial poultry farms. For more details on number, density and type of poultry farms, see [3]. Most poultry farms are concentrated in an area in the central part of the country (‘Gelderse Vallei’) and this DPPA had a density of 0.97 poultry farms per km^2^ in 2008.

### Description of the VPI compartment

**Fig 2:**
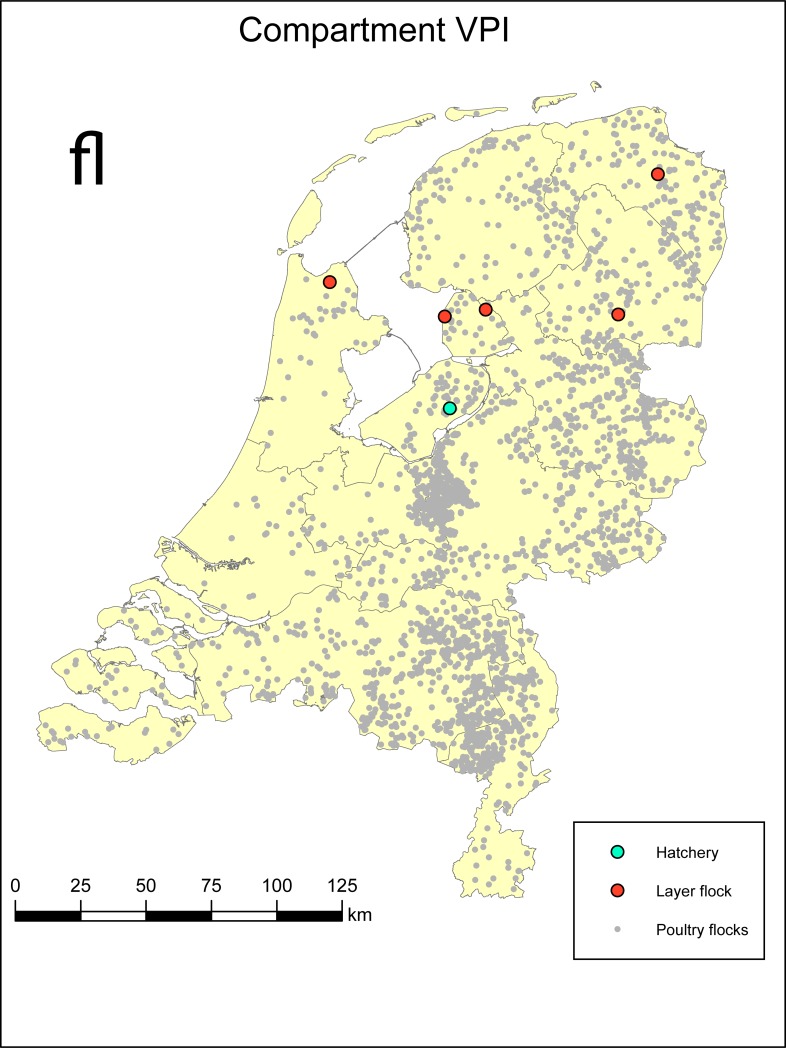
The location of the 5 layer farms and the hatchery of the compartment of VPI and that of other commercial poultry farms in the Netherlands (database of 2008, representative of the situation when the status of the example compartment VPI was granted and discussed in the Netherlands). For more details on number, density and type of poultry farms in 2008, see [3]. The DPPA in the central part of the country has a density of 0.97 poultry farms per km^2^ (Gelderse Vallei).

The VPI compartment consists of at most five layer farms and one hatchery. The location of these farms and that of all poultry farms in the Netherlands is depicted in Fig 2. Part of the definition of the VPI compartment is that any one of the farms that becomes part of an HPAI surveillance zone (10 km zone) is taken out of the VPI compartment. The hatchery of VPI is solely dedicated to producing fertilized chicken eggs and does not have live animals (chickens) on its premise. There is only transport of eggs between the compartment farms (supplying the hatchery), i.e. between the compartment farms there is no transport of live animals. Fertilised eggs are transported from all five layer farms to the hatchery. From there, 10 day old incubated eggs are transported to France and Germany, to be used for the production of vaccines against human influenza.

VPI developed a detailed biosecurity plan; important elements include quality assurance schemes, procedures for animal and human movement control, poultry health measures (including vaccinations, medications and other veterinary care), control over vehicles, security of feed and water sources, and control of pests and wild bird populations. For a detailed list of biosecurity measures for compartments, see [8]. Direct contact processes between VPI farms consist of egg transports, and indirect contact processes are of the following type: feed deliveries, person visits and collection of rendering material. The biosecurity arrangements surrounding these four processes are as follows:

Eggs are transported twice per week from each layer farm to the hatchery, by one-to-one transport between layer farm and hatchery. There is one truck for all egg transports within the compartment, and this truck is not used for transport of eggs stemming from non-compartment farms. Also, all egg trays are only used within the compartment. The truck leaves disinfected and empty from the hatchery to a layer farm, where the eggs are uploaded, and the truck is disinfected again before departure from the layer farm to the hatchery. After the eggs are delivered at the hatchery, the truck is disinfected again.

Each Monday one transport truck with animal feed from outside the compartment visits all farms within the compartment, and then leaves the compartment again (so at a frequency of once per week). After leaving a farm, the wheels and wheel arches of the truck are disinfected.

All farms of the compartment are being visited by people throughout the first half of the week, at a frequency of once every 2 weeks. A veterinarian visits all farms of the compartment on a Monday, at a frequency of once per 3 weeks. A sample taker visits all farms of the compartment on a Monday, at a frequency of once per 15 weeks. Showering before and after each farm visit and wearing corporate clothing during a visit is compulsory for the technical advisor, veterinarian and sample taker.

To collect rendering material, a truck from outside the compartment visits all farms of the compartment on a Monday at a frequency of once per 4 weeks. After leaving a farm, the truck is disinfected.

### Parameter values used in the model for the VPI compartment

For the analysis of the VPI compartment specifically, two transmission pathways in the generic model were set to 0: VPI does not transport live chickens from the compartment to farms outside the compartment, and VPI does not transport live chickens between farms within the compartment.

Table 1 summarizes the type of between-farm contacts within the VPI compartment, the frequency of these contacts in the compartment as described in the VPI biosecurity plan, and the probability of HPAI transmission per contact type. The latter were estimated from the H7N7 HPAI epidemic in the Netherlands in 2003 by Ssematimba et al. [14]. Because one and the same truck is used for egg transport in the VPI compartment, each farm has contact with another farm twice a week by means of egg transport via the hatchery. As a worst-case estimate of HPAI transmission, and because the effect of biosecurity measures like truck disinfection on virus transmission in practice has never been quantified, we first ignore in our analysis the effect of biosecurity measures executed by VPI in addition to the measures taken in the field in 2003. Furthermore, as survival of the HPAI virus in the environment during at least a few days is very well possible, it is assumed that contacts between farms with the same truck are still infectious over a week (see [15]).

**Table 1:** Overview of between-farm contact types within the VPI compartment, their frequency, and the probability of HPAI transmission per contact, as estimated from the H7N7 HPAI epidemic in the Netherlands in 2003. By multiplication of these two data, the transmission rate was calculated.

Type of Contact	# contacts per day	Probability of HPAI transmission per contact ^1)^	Transmission Rate (per day)
Egg transport ^2)^	2/7	0.308	0.088
Feed delivery	1/7	0.0414	0.0059
Professional contact	(1/2+1/3+1/15)/7 ^3)^	0.133	0.017
Rendering Contact	(1/4)/7	0.246	0.0088
Animal transport	0	1 ^4)^	0

^1)^ From Ssematimba et al. [14]. The biosecurity measures executed by VPI in addition to the measures taken in the field in 2003 are not incorporated in these probabilities (worst-case). It is assumed that contacts between farms with the same truck are still infectious over a week.

^2)^ Each farm has contact with another farm twice a week by means of egg transport via the hatchery.

^3)^ Technical supervisor: 1 per 2 weeks; veterinarian 1 per 3 weeks; sampler 1 per 15 weeks.

^4)^ Assumed probability of transmission of 1 (worst-case).

Most contacts between farms in the VPI compartment take place in one or a few days, usually on a Monday or during the first half of the week. This implies that infectious material attached to a person or a vehicle stays infectious during that short period of one or a few consecutive days. Again, as a worst-case scenario we ignore disinfection measures executed by VPI in the analysis (as far as these are more effective than the measures carried out across the HPAI outbreak areas in 2003). So it is assumed that upon infection a transport remains infectious during the full weekly period of transport within the VPI compartment.

Specific transportation routes which are repeated every week within the VPI compartment (trucks from farm A to B and from B to C etc.) were not known to us, and thus fixed contact structures among farms in the compartment were not incorporated in the analysis. We assumed random mixing where each farm has the same probability to infect another farm within the compartment.

We note that several of the contact event types that we included as specific for the compartment, and thereby contributing to the probability *p*_c_, are in fact also allowed outside the compartment, so they are not an extra allowed contact: feed delivery, professional contact and rendering contact. However, because of the closed contact structure within the compartment (designed to minimize the risk of introduction of HPAI into the compartment), these contacts always occur between the same compartment farms, unlike the situation for farms outside the compartment, where these contacts occur to ‘random’ other farms and are thus taken into account through the transmission kernel *p*_0_.

### Sensitivity analysis of biosecurity measures executed by VPI

The probabilities of HPAI transmission (Table 1) were estimated from the H7N7 HPAI epidemic in the Netherlands in 2003 [14]. Thus, these probabilities represent the biosecurity which surrounded the contact events between poultry farms at that time, i.e. any possible additional biosecurity realized through the measures taken by VPI to maintain their certified compartment status are not incorporated. Thus, these transmission probabilities are worst-case estimates. Unfortunately, quantitative data on the effect of biosecurity measures such as disinfection (of trucks, people etc.) are not available. To evaluate the effect of hypothetical improvements of biosecurity in comparison to the 2003 reference, a sensitivity analysis was carried out in which the probability of transmission of *each* of the pathways of Table 1 was reduced separately with 10%, 50% and 90%. The effect was also evaluated when *all* probabilities were reduced at the same time.

## Results

### Transmission risk from farms in the VPI compartment

The VPI compartment serves as an example of a compartment with only egg transports, i.e. no animal transports. When using the kernel parameter values from Boender et al. [13] and compartment parameters as presented in Table 1, the probability of HPAI transmission between two farms in the compartment amounted to *p*_c_ = 0.59 (calculated using Eq 4). So when, despite all biosecurity measures, HPAI would be introduced on one of the farms of the VPI compartment, the probability of infecting any one of the other farms of the compartment is high.

The between-farm transmission probability *p*_c_ of 0.59 is mainly due to the allowed contact events between compartment farms; the contribution of neighbourhood transmission is much smaller. For this reason the approximation of Eq 7 applies. With *p*_c_ = 0.59 and *N*_c_ = 5 farms with live chicken for the VPI compartment, the difference in the between-farm reproduction number between the situation with and without compartment equals Δ*R*_0,*m*_ = 2.36 for each of the compartment farms. If the same VPI farms were *not* part of a poultry compartment, i.e. when during an HPAI epidemic the between-compartment farm contacts of Table 1 are all prohibited, the *R*_0,*m*_ would be close to 0.05 for each of the VPI farms (calculated using Eqs 3 and 4, setting ∑_*l*_*h*_*l*_ = 0).

### Transmission risk for a compartment including chicken transport

Now a fictitious compartment of the same size and contact frequencies as VPI is considered, but with an additional contact of 1 animal transport per month between two arbitrary farms within the compartment. This transport frequency of live chicken was chosen to reflect transport from rearing layer farms to layer farms, and leads to a frequency of 1 per 120 days that a farm will have contact with all other 4 farms. This yields a value of *p*_c_ = 0.62, based on using Eq 4 with parameter values of Table 1 with a contact rate by animal transport of 1/120 instead of 0. With *N*_c_ = 5 for the size of the compartment we obtain Δ*R*_0,*m*_ = 2.48, again using Eq 7. This risk difference is not very different from that calculated for the VPI compartment, because of the relatively low frequency of contact by animal transport compared to that of the other contacts of Table 1.

### Risk of jumps of HPAI between areas via the VPI compartment

Here the VPI compartment serves as an example of a compartment with only egg transports, i.e. no animal transports, and of a compartment with no compartment farm situated in a DPPA. The risk of jumps of HPAI from one area to another, due to the compartment, was evaluated by calculating the ratio between (1) the probability of jumps caused by ‘single-event’ transmission and (2) the probability of jumps caused by ‘triple-event’ transmission via farms of the compartment. For the interpretation of this value, see the M&M section. In Table 2 the results for the case of VPI are given.

**Table 2:** The risk of HPAI transmission jumps from one area to another, due to a compartment with only egg transports (i.e. no animal transports), and with no compartment farms situated in a DPPA.

Quantity	Value
Ratio^1)^	0.0015 (0.0013–0.0017)
‘Single-event’ transmission probability^2)^	1.2 10−6–1.6 10^−5^
‘Triple-event’ transmission probability^2)^	3.6 10−10–5.1 10^−8^

^1)^ The risk of jumps is expressed as mean ratio of ‘triple-event’ transmission via the compartment and ‘single-event’ transmission by neighbourhood transmission (the 5%-95% interval given between brackets).

^2)^ The range (min-max) of the probability for ‘single-event’ transmission and for ‘triple-event’ transmission are added.

As shown in Table 2, the ‘triple-event’ transmission via farms of the VPI compartment is 0.0015 times smaller than the ‘single-event’ transmission from a non-compartment farm in a DPPA (here Gelderse Vallei) to a non-compartment farm outside this DPPA. Furthermore, this is a worst-case scenario, because biosecurity measures ‘additional to the 2003 average’ executed by VPI in the compartment were ignored in this analysis. In reality, this ratio will thus be even smaller.

### Risk of jumps of HPAI between areas via a ‘VPI-like’ compartment with one farm in a DPPA

The risk of jumps was also evaluated for a fictitious compartment of the same size and contact frequencies as VPI, but with one farm located in the DPPA (here Gelderse Vallei) as depicted in Fig 1B. The results for these jump probabilities are listed in Table 3.

**Table 3:** The risk of HPAI transmission jumps from one area to another, due to a compartment with one farm located in the DPPA.

Quantity	Value
Ratio^1^^)^	0.57 (0.46–0.69)
‘Single-event’ transmission probability^2)^	1.1 10−6–0.010
‘Triple-event’ transmission probability^2)^	1.2 10−7–8.9 10^−5^

^1)^ The risk of jumps is expressed as mean ratio of ‘triple-event’ transmission via the compartment and ‘single-event’ transmission by neighbourhood transmission (the 5%-95% interval given between brackets).

^2)^ The range (min-max) of the probability for ‘single-event’ transmission and for ‘triple-event’ transmission are added.

In this case the ‘triple-event’ transmission via farms of the fictitious compartment adds about 50% (ratio of 0.57) to the risk caused by the ‘single-event’ transmission from a compartment farm now located in the DPPA to a non-compartment farm outside the DPPA. Thus, when one farm of the compartment is located in the DPPA, the ratio is much higher, i.e. the risk of jumps of HPAI via the compartment farms becomes much more important.

### Risk of jumps of HPAI between areas via a compartment including chicken transport and with one farm in a DPPA

Now a fictitious compartment of the same size and contact frequencies as VPI is considered, with one farm in the DPPA (here Gelderse Vallei) and with transport of animals (chicken) and eggs. The frequency is again 1 animal transport contact per month between two arbitrary farms within the compartment, leading to a frequency of 1 per 120 days that a farm will have contact with all other 4 farms. In this case we find *p*_c_ = 0.61, and the jump probabilities are listed in Table 4.

**Table 4:** The risk of HPAI transmission jumps from one area to another, due to a compartment with one farm located in the DPPA and with transport of animals (chicken) and eggs.

Quantity	Value
Ratio^1)^	0.62 (0.46–0.78)
‘Single-event’ transmission probability^2)^	1.1 10−6–0.0020
‘Triple-event’ transmission probability^2)^	1.2 10−7–1.2 10^−4^

^1)^ The risk of jumps is expressed as mean ratio of ‘triple-event’ transmission via the compartment and ‘single-event’ transmission by neighbourhood transmission (the 5%-95% interval given between brackets).

^2)^ The range (min-max) of the probability for ‘single-event’ transmission and for ‘triple-event’ transmission are added.

The ‘triple-event’ transmission via farms of this fictitious compartment again adds about 50% (ratio of 0.62) to the risk caused by the ‘single-event’ transmission from a compartment farm now located in the DPPA to a non-compartment farm outside the DPPA. In other words, the total transmission probability of a jump from one area to another is increased with about 50% compared to that of the ‘always occurring background’ single-event transmission. When one farm of the compartment is located in the DPPA, and transport of live chicken occurs within the compartment, the ratio is similar as above in Table 3. Thus, the *location* of the compartment farm (whether or not in the DPPA) is most important, and not the transport of live chicken compared to that of eggs, due to its lower transport frequency.

### Sensitivity analysis of biosecurity measures carried out by VPI

Table 5 shows the reduction in reproduction number *R*_0,*m*_ of the VPI compartment farms when the transmission probability of a specific transmission pathway within the compartment was reduced with 10%, 50%, and respectively 90%, as potential effect of biosecurity measures executed by VPI. When transmission of HPAI by egg transports can be reduced with 90% (i.e. a factor 10 reduction) by biosecurity measures such as disinfection of trucks and egg trays, the reproduction number *R*_0,*m*_ will be reduced with 54%. With such a biosecurity improvement, corresponding to much better biosecurity than in 2003, the reproduction number is reduced to a value of 1.1, still leaving it at a much higher value than the value of 0.05 for a non-compartment farm at the same location. We note that even in the fictitious case of 100% biosecurity for the egg transports, the reproduction number remains much larger than 0.05, namely 0.9, if the biosecurity improvement is applied to the egg transports only. According to Table 5, egg transports are the most important pathway of HPAI transmission among farms within the compartment, due to its high transmission probability and high transport frequency (see Table 1). Focussing biosecurity measures on one contact type only leads to a partial reduction of *R*_0,*m*_.

**Table 5:** Relative reduction (in %) of reproduction number *R*_0,*m*_ of the VPI compartment farms as function of the reduction in transmission probability (10, 50, 90%) of a specific route, due to biosecurity measures within the compartment.

Type of Contact	10%	50%	90%
Egg transport	4.6	26	54
Professional contact	0.9	4.4	8.1
Rendering Contact	0.4	2.2	4.0
Feed delivery	0.3	1.5	2.7

**Fig 3:**
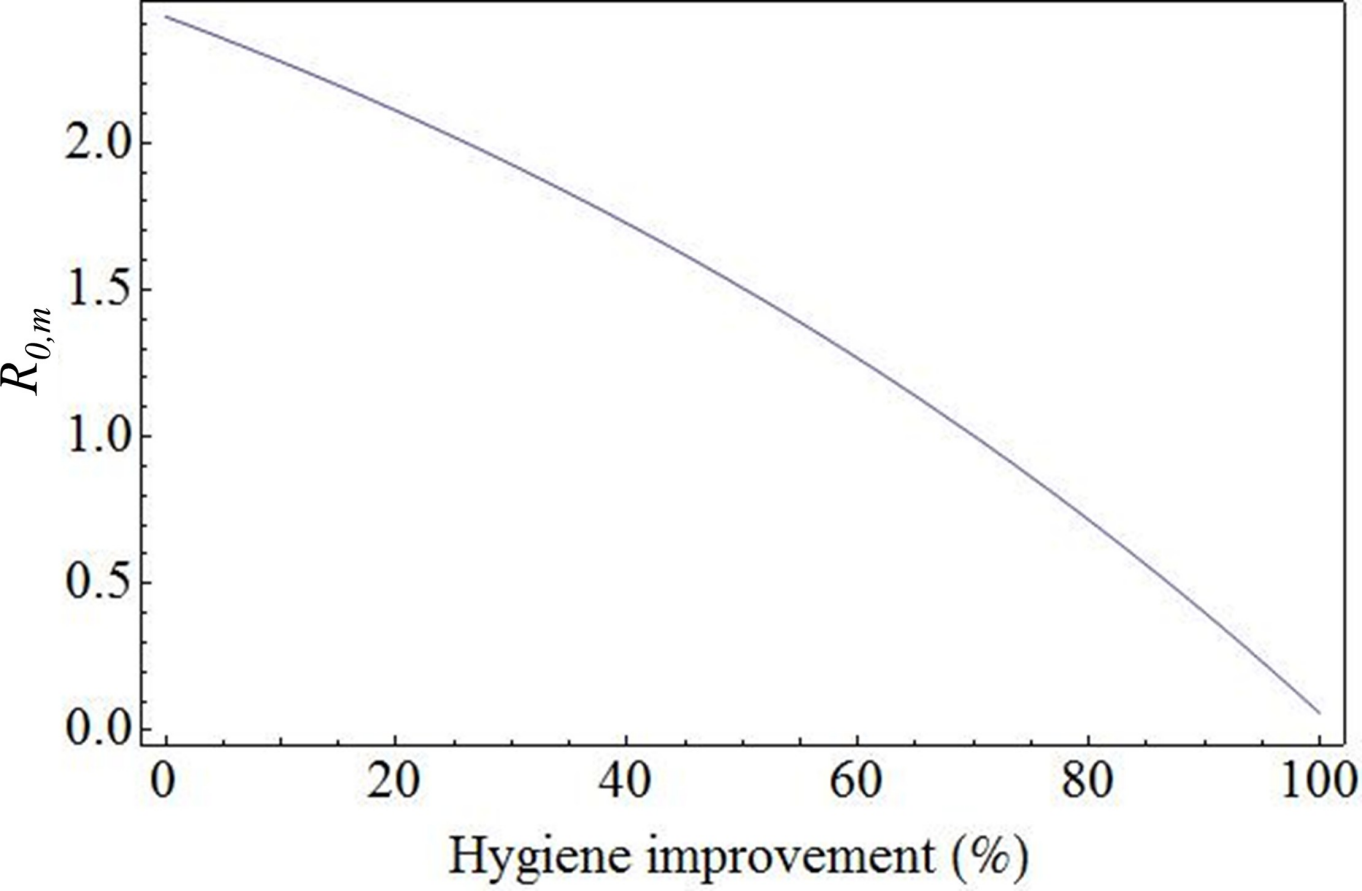
Reproduction number *R*_0,*m*_ of VPI compartment farms, when *all* transmission probabilities of the contact types of Table 1 are reduced with a certain percentage, due to improvement of biosecurity measures within the compartment. A biosecurity improvement of 0% represents the biosecurity status of poultry farms during the HPAI epidemic in the Netherlands in 2003. A biosecurity improvement of 100% is a purely fictitious scenario of perfect biosecurity leading to zero risk.

Fig 3 shows the reduction in reproduction number *R*_0,*m*_ of the VPI compartment farms, when *all* transmission probabilities of the contact types of Table 1 were reduced with a certain percentage, due to improvement of biosecurity measures within the compartment. A biosecurity improvement of 0% represents the biosecurity status of poultry farms during the HPAI epidemic in the Netherlands in 2003. To reach an *R*_0,*m*_ for the compartment farms that is only 0.10, i.e. only approximately twice as large as that for the non-compartment farms, a biosecurity improvement of 98% is needed for all contact types, corresponding to a factor 50 reduction by biosecurity measures like disinfection.

### Effect of distance of a compartment to a DPPA

**Fig 4:**
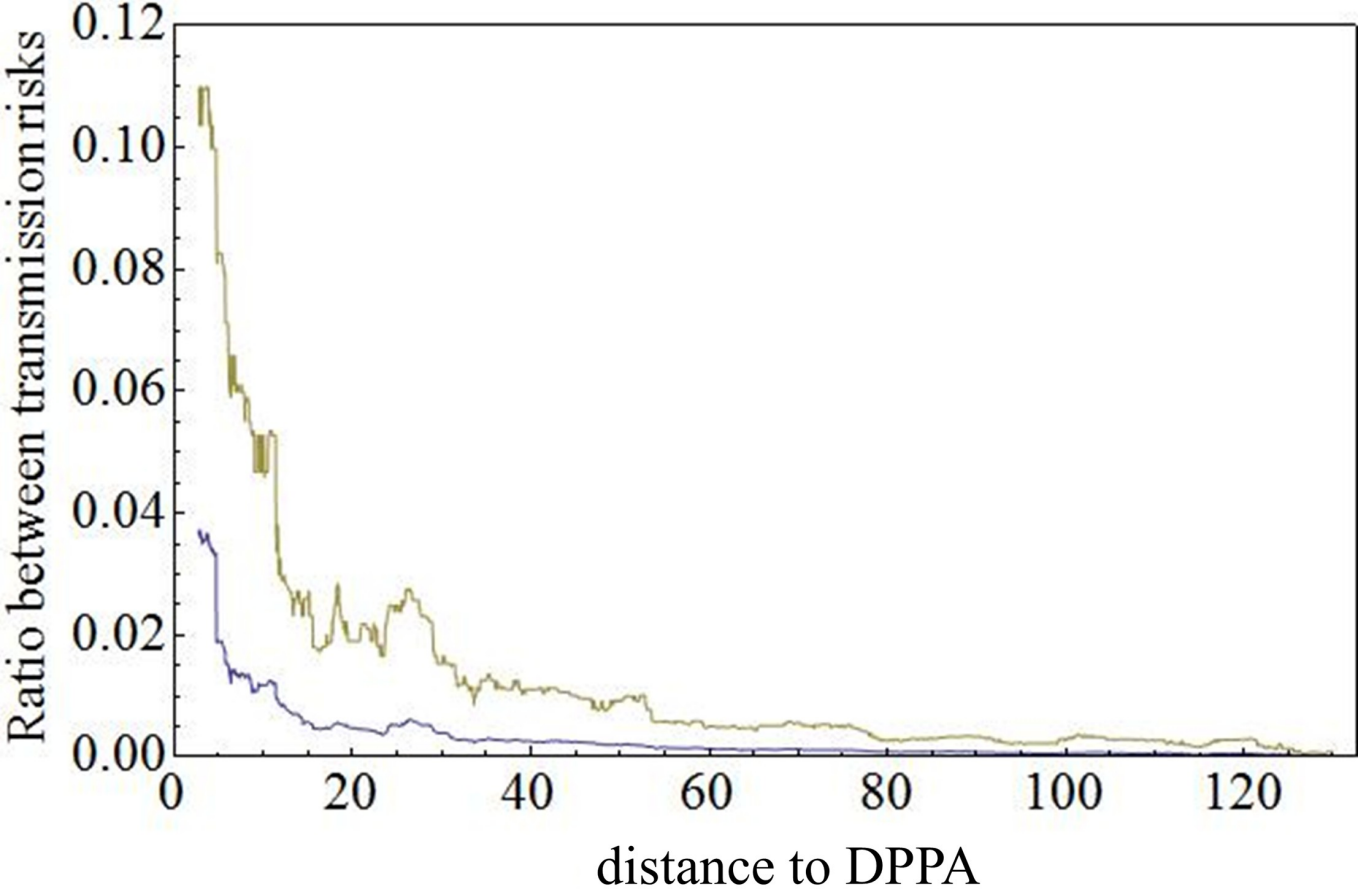
Risks of HPAI transmission jumps expressed as ratio between ‘triple-event’ transmission via the compartment and ‘single-event’ transmission, as function of the distance of the compartment farm to the nearest farm in the DPPA (here Gelderse Vallei, in the central part of the Netherlands). Shown is a rolling average plot calculated with an averaging window of 5 km., based on a set of 10,000 pairs {distance, ratio} (see M&M section). The rolling average of the mean (blue line) and 95% percentile (green line) are presented. The 5% percentile is equal to 0 throughout.

Fig 4 shows the risks of HPAI transmission jumps expressed as ratio between ‘triple-event’ transmission via the compartment and the ‘single-event’ transmission as a function of the distance of a compartment farm to the nearest farm in the DPPA (here Gelderse Vallei). Fig 4 shows that if one of the compartment farms is located in or close to the DPPA, the risk of HPAI transmission jumps via the compartment cannot be neglected anymore. It increases about tenfold when the distance to the DPPA is reduced from 40 km to 0 km, reaching a value that is 1.5–1.6 times larger than the jump risk in absence of a compartment in the country. The mean ratio for the VPI compartment was 0.0015 (see Table 2), and this low value is due to the large distance of approximately 60 km of VPI to the DPPA.

### Effect of compartment size (number of farms)

Using Eq 7, the reproduction number *R*_0,*m*_ of compartment farms increases linearly (almost proportionally) with the number of farms *N*_c_ (housing live chicken) in that compartment. This relationship is based on the assumption that all farms in the compartment have random contacts with each other. This increase in risk could be avoided by introducing separate networks within the poultry compartment: for example when comparing a compartment size of 10 to that of 5 by using two trucks for egg transport, thus connecting only half of the farms by one truck and the other half of the farms by the other truck. For each of these specific situations, the generic model developed in this study can be adapted and used to evaluate the corresponding risks.

## Discussion and conclusions

In the current study two risks were evaluated, comparing the situations with and without presence of a poultry compartment in the Netherlands: (1) the local HPAI transmission risk from farms within the compartment, and (2) the risk of a ‘jump’ of HPAI via the compartment between different areas in the country. Gemmeke et al. [10] proposed the use of a HACCP risk analysis system to determine the effectiveness of the biosecurity plan of a compartment. In order to assess transmission risks within a compartment as well as between compartment farms and non-compartment farms, epidemiological analysis of the between-farm contact structure is required. Nickbakhsh et al. [16] was the first to perform such an analysis, evaluating and comparing zoning and compartmentalisation of UK poultry industry components in the context of HPAI transmission risks. They quantified these transmission risks, using data for the contact network between poultry farms, slaughterhouses and catching companies in the UK to define network mediated links and using spatial proximity of farms to define hypothetical ‘spatially mediated’ links. Where Nickbakhsh et al. [16] were interested in the transmission risks in the absence of a movement ban, our analyses consider the risks in the period after the imposition of movement restrictions. The reason for this focus is that most of the epidemic spread during the 2003 HPAI epidemic in the Netherlands took place after the first outbreak was detected and movement restrictions were put in place.

Our model uses information on the contact network between the compartments farms, in combination with estimated probabilities of HPAI transmission per contact type from Ssematimba et al. [14] to quantify the transmission risks between compartment farms. To evaluate the transmission risks between compartment farms and non-compartment farms the model uses a spatial transmission kernel [13] that describes the observed between-farm transmission in 2003 in the presence of movement restrictions.

The main conclusions based on our analyses are as follows. Firstly, granting compartment status to a set of farms with egg transports only leads to a substantial increase of the between-farm reproduction number *R*_*0*,*m*_ between these farms as compared to a situation where these farms would be under the same movement restrictions as other farms during an epidemic. In the specific example presented, the *R*_*0*,*m*_ of the compartment farms increases by a factor 50 from 0.05 (when not part of a poultry compartment) to 2.4 (when part of a poultry compartment). The relatively high transmission risk within the compartment arises as a result of the closed contact structure designed to keep HPAI introduction risks from outside the compartment as low as possible. This implies that if one compartment farm is suspected of being infected with HPAI, all other farms of the compartment must be considered as dangerous contact farms. This finding supports the rationale of VPI policy to remove a compartment farm from the compartment as soon as it becomes part of a surveillance zone (10 km ring) around a detected non-compartment farm.

Secondly, or analysis suggests that the risk of jumps of HPAI from one area via a poultry compartment to another area in the country is negligible for the example of the compartment with egg transports only. The reason for this low risk is the long distance of approximately 60 km between the VPI farms and the nearest DPPA (Densely Populated Poultry livestock Area), which is the Gelderse Vallei in the central part of the Netherlands \. Thus, the approval in 2011 of the compartment status for VPI is supported by the current quantitative study.

If one of the compartment farms is located in or close to a DPPA, the risk of HPAI transmission jumps via the compartment cannot be neglected anymore. It increases about tenfold when the distance to the DPPA is reduced from 40 km to 0 km, reaching a value that is 1.5–1.6 times larger than the jump risk in absence of a compartment in the country.

The between-farm risks were quantified using a worst-case assumption for the effectiveness of biosecurity measures, namely taken equal to that during the HPAI epidemic in the Netherlands in 2003. Additional biosecurity that might be achieved by a strict adherence to the biosecurity plan of the compartment (as listed in [8]) was ignored in our default calculations, as the quantitative effect of each of these measures on virus transmission in practice is still unknown. Instead, the effect of hypothetical improvements in biosecurity as compared to the 2003 reference was evaluated in a sensitivity analysis, with the following results. Focussing the biosecurity measures within the poultry compartment VPI on the transport of eggs yields the strongest reduction of the between-farm reproduction number *R*_*0*,*m*_ of the example compartment. This is due to the high frequency of these transports in this compartment. But even when HPAI transmission by egg transports was fully blocked by biosecurity measures, the *R*_*0*,*m*_ of the VPI farms is reduced by 63% to a value of 0.9, still much higher than the mean *R*_*0*,*m*_ of farms in the same location not being part of a compartment (0.05). Thus, in order to reduce risks further, the biosecurity measures must be aimed at *all* contact possibilities together between farms within the compartment, like egg transports, feed delivery, professional contacts and rendering contacts.

Our analyses also gives some insight in how a compartment influences the risk of HPAI jumps between different areas within the country. From this study a number of strategies emerge for limiting this risk, other than the obviously important strategy of applying stringent biosecurity measures to all contact events. These strategies could be considered as or translated into requirements to criteria that should be met by applicants in order to obtain compartment status. These strategies are as follows: (1) avoiding the inclusion in a compartment of farms located in/or close to a DPPA; (2) setting the transport/visitor route(s) such that compartment farms located closest to DPPA/high-risk areas are visited last on the day.

Finally, the closed contact structure of a compartment is an aspect that deserves close scrutiny. As a consequence of this contact structure, each farm within the compartment has frequent (indirect) contacts to all other farms, yielding ample HPAI transmission possibilities. The importance of strict biosecurity measures applied to vehicles and professionals going from one compartment farm to the next has been confirmed in this study. Also, any options for reducing the number of connections in the contact network deserves to be scrutinized. One strategy could be to aligning all regular transport and visitor routes as much as possible to one fixed route along the compartments farms. Another strategy of interest is to set transport routes such that farms located closest to DPPA/high-risk areas are visited last on the day. Finally, the risk could be reduced by maintaining two or more separated transport sub-networks within the poultry compartment. For each of these specific situations, the generic model developed in this study can be adapted and used to evaluate the corresponding risks. Although our model parameter estimates are based on Dutch outbreak data and therefore no immediate extrapolation to other countries is possible, our approach and general results may still benefit the evaluation of compartments in other countries.
