# Correction: Risk of poultry compartments for transmission of Highly Pathogenic Avian Influenza

**DOI:** 10.1371/journal.pone.0212986

**Published:** 2019-02-22

**Authors:** The PLOS ONE Staff

There are multiple formatting errors in Table 2. Please see the complete, correct table here.

**Table 2:** The risk of HPAI transmission jumps from one area to another, due to a compartment with only egg transports (i.e. no animal transports), and with no compartment farms situated in a DPPA.

Quantity	Value
Ratio^1^^)^	0.0015 (0.0013–0.0017)
‘Single-event’ transmission probability^2^^)^	1.2 10^−6^–1.6 10^−5^
‘Triple-event’ transmission probability^2^^)^	3.6 10^−10^–5.1 10^−8^

^1)^ The risk of jumps is expressed as mean ratio of ‘triple-event’ transmission via the compartment and ‘single-event’ transmission by neighbourhood transmission (the 5%-95% interval given between brackets).

^2)^ The range (min-max) of the probability for ‘single-event’ transmission and for ‘triple-event’ transmission are added.

There are multiple formatting errors in Table 3. Please see the complete, correct table here.

**Table 3:** The risk of HPAI transmission jumps from one area to another, due to a compartment with one farm located in the DPPA.

Quantity	Value
Ratio^1^^)^	0.57 (0.46–0.69)
‘Single-event’ transmission probability^2^^)^	1.1 10^−6^–0.010
‘Triple-event’ transmission probability^2^^)^	1.2 10^−7^–8.9 10^−5^

^1)^ The risk of jumps is expressed as mean ratio of ‘triple-event’ transmission via the compartment and ‘single-event’ transmission by neighbourhood transmission (the 5%-95% interval given between brackets).

^2)^ The range (min-max) of the probability for ‘single-event’ transmission and for ‘triple-event’ transmission are added.

There are multiple formatting errors in Table 4. Please see the complete, correct table here.

**Table 4:** The risk of HPAI transmission jumps from one area to another, due to a compartment with one farm located in the DPPA and with transport of animals (chicken) and eggs.

Quantity	Value
Ratio^1^^)^	0.62 (0.46–0.78)
‘Single-event’ transmission probability^2^^)^	1.1 10^−6^–0.0020
‘Triple-event’ transmission probability^2^^)^	1.2 10^−7^–1.2 10^−4^

^1)^ The risk of jumps is expressed as mean ratio of ‘triple-event’ transmission via the compartment and ‘single-event’ transmission by neighbourhood transmission (the 5%-95% interval given between brackets).

^2)^ The range (min-max) of the probability for ‘single-event’ transmission and for ‘triple-event’ transmission are added.

The publisher apologizes for these errors.
